# Senolytic Treatment With Dasatinib and Quercetin Reshapes Influenza‐Specific CD8 T Cell Responses During Infection in Aged, Vaccinated Mice

**DOI:** 10.1111/acel.70345

**Published:** 2025-12-29

**Authors:** Andreia N. Cadar, Blake L. Torrance, Sofie A. Fischler, Darlene A. Djaba, Zena Haddad, Dominique E. Teskey, Nagaraju Marka, Kelsey A. Gorgei, Erica C. Lorenzo, Laura Haynes, Jenna M. Bartley

**Affiliations:** ^1^ Center on Aging UConn Health Farmington Connecticut USA; ^2^ Department of Immunology University of Connecticut School of Medicine Farmington Connecticut USA; ^3^ Chemistry & Biochemistry Department Nazareth University Rochester New York USA; ^4^ Department of Molecular and Cell Biology University of Connecticut Storrs Connecticut USA

**Keywords:** aging, infection, influenza, senolytic, T cells, vaccination

## Abstract

Older adults are disproportionately affected by infectious diseases like influenza (flu) due to immune declines and poor vaccine responses. Senolytics have been shown to improve various age‐related conditions and positively influence infection outcomes, yet their potential to enhance vaccine responses has not yet been explored. Here, we evaluated the potential of senolytic combination Dasatinib (D) and Quercetin (Q) treatment prior to influenza vaccination to potentiate immune responses in aged mice. D + Q had minimal impact on overall vaccination and flu outcomes in vaccinated mice, including viral load and lung pathology. However, we observed altered CD8 T cell immunodominance and increased serum total PR8 (whole flu) IgG antibodies in D + Q treated vaccinated aged mice during infection. These findings reveal a new aspect of immunomodulation with senolytics.

## Graphical abstract

Impaired vaccination responses leave older adults more vulnerable to severe influenza (flu) infections. Pretreatment with senolytic Dasatinib and Quercertin (D + Q) did not improve vaccine responses but altered some responses to sublethal flu challenge. Despite these changes, overall flu outcomes were not impacted.

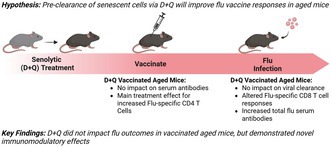

## 1 Introduction

Aging leads to progressive declines in immune responses, which have been shown in both murine and human studies (Lefebvre et al. 2012; Lefebvre, Lorenzo, et al. 2016; Ukraintseva et al. 2021). By 2060, nearly 1 in 4 Americans will be 65 years or older and the older population will surpass the population of children under 18 years old (United States Census Bureau, n.d.). Age‐related declines in immune responses lead to increased susceptibility and severity of infectious diseases. Respiratory infections, such as COVID‐19 and influenza (flu), disproportionately impact older adults. Indeed, older adults have increased risk for hospitalization and death due to flu infection, with approximately 90% of flu‐related deaths occurring in those over 65 years old (Thompson et al. 2003). Vaccination remains the most effective defense against flu and other infectious diseases; however, impaired immune responses with aging also reduce vaccine‐induced protection. Specifically, in terms of flu vaccination, older adults have reduced antibody titers, impaired B cell responses, reduced T cell proliferation, and overall decreased protection from flu infection (Murasko et al. 2002). FDA‐approved flu vaccines for older adults, such as the high dose or adjuvanted flu vaccine, consistently improve protective responses compared to the standard vaccine in older adults (Ng et al. 2019), however, the burden of flu remains high in older adults, suggesting current vaccination strategies are still inadequate. Robust vaccination responses require the coordination of multiple cell types, signaling pathways, and cell trafficking to generate strong cell‐mediated and humoral responses. Thus, it's likely that targeting specific immune deficits is not enough to overcome the totality of age‐related impairments.

Tissue microenvironments and their interactions with various immune cells are critical in driving robust immune responses. It has been well established that with age, chronic, low‐grade, and sterile inflammation, termed inflammaging, contributes greatly towards many age‐related declines, including that of the immune system (Franceschi et al. 2018). We and others have also demonstrated that the aged microenvironment negatively impacts CD4 T cell differentiation and function (Lefebvre et al. 2012; Lorenzo et al. 2022). More specifically, during flu infection and vaccination, aged mice have increased regulatory T cells (Tregs) and T follicular regulatory cells (Tfr), as well as dysfunctional T follicular helper cells (Tfh), leading to impaired germinal center formation and reduced antibody production (Lefebvre, Lorenzo, et al. 2016; Lefebvre, Masters, et al. 2016). Adoptive transfer experiments have shown that young CD4 T cells have similar deficits when transferred into an aged host (Lefebvre et al. 2012), suggesting the aged microenvironment contributes to impaired function. Similarly, B cell function is impacted negatively by the aged inflammatory microenvironment, with increased age‐associated B cells, impaired germinal center formation and class‐switch recombination, among other deficits (Frasca et al. 2020; Lee et al. 2023). Cytotoxic CD8 T cell responses, which are essential to flu responses, are also impaired in the aged microenvironment, with reduced proliferation and cytotoxicity (Jiang et al. 2009). Recent research has pointed to cellular senescence as a culprit behind altered tissue microenvironments with aging and correspondingly is a major driver of aging physiology.

Cellular senescence, as first described by Hayflick and Moorhead, is a mostly irreversible state of proliferation arrest that occurs as a response to cellular stress (Nakagami 2020). Despite experiencing proliferative arrest, these cells remain metabolically active and secrete a pro‐inflammatory signature termed senescence associated secretory phenotype (SASP) (Coppé et al. 2010; Kumari and Jat 2021). Age‐related accumulation of senescent cells has been shown to have a causal role in frailty (Xu et al. 2015), declines in physical function (Xu et al. 2018), and age‐related bone loss (Farr et al. 2017) among other disorders. However, research on the role of senescence in immune responses is less clear. While some have demonstrated that senolytics can improve immune responses to certain infections, such as Mouse Hepatitis Virus and SARS‐CoV‐2 infections in both brain organoids and mouse models (Aguado et al. 2023; Camell et al. 2021; Pastor‐Fernández et al. 2023), we have previously reported minimal benefits of Dasatinib (D) and Quercetin (Q) senolytic treatment in overall flu outcomes (Torrance et al. 2023) despite improved CD4 T cell differentiation (Lorenzo et al. 2022). Nonetheless, the utility of senolytics to improve vaccine responses with aging has not yet been explored. Thus, here, we aimed to determine the effect of senolytic D + Q treatment on flu vaccination responses in aged mice, as well as further probe the effectiveness of the vaccine‐induced protection with subsequent flu challenge. We anticipated that senolytics would support more youthful ratios of CD4 T helper cell subsets to enhance vaccination responses. Our findings, however, show that while D + Q treatment prior to vaccination in aged mice did not potentiate vaccination responses, CD8 T cell immunodominance hierarchy was influenced during sublethal flu challenge and de novo responses to infection were potentiated.

## 2 Results

### 2.1 Effects of D + Q on Vaccine‐Induced Serum IgG and CD4 T Cell Responses

**FIGURE 1:**
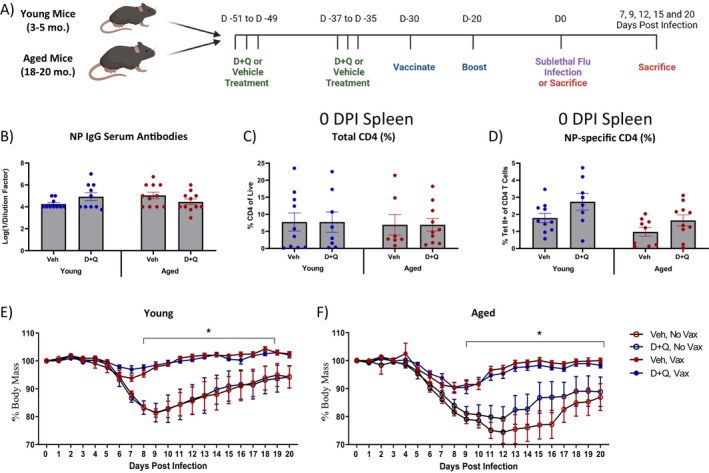
Experimental design and evaluation of the impact of D + Q treatment prior to vaccination in young and aged mice. Young (3–5 months old) and aged (18–20 months old) C57BL/6JN (B6) male mice were treated with senolytic combination dasatinib plus quercetin (D + Q) or vehicle control prior to being vaccinated and boosted with flu nucleoprotein (NP) with alum or PBS with alum control. Mice were subsequently infected with a sublethal dose of PR8 H1N1 influenza. (A) Experimental design. (B) NP IgG Serum antibodies were examined 30 days post initial vaccination by ELISA from serum collected via cardiac puncture. (C, D) Total and NP‐specific splenic CD4 T cells were evaluated 30 days post initial vaccination by flow cytometry via flu NP MHC II tetramer staining. (E, F) Percent of original weight loss was measured throughout sublethal PR8 infection in young and aged mice. * indicates statistical significance between unvaccinated and vaccinated mice regardless of treatment at various DPI. Data are presented as mean ± standard error of the mean (SEM). Two‐way ANOVA was performed, followed by Šidák's test for multiple comparisons. Results were considered significant at *p* < 0.05. *N* = 9–10/group.

To assess the impact of senolytics on vaccine responses, young (3–5 months) and aged (18–20 months) male mice were treated with D + Q via oral gavage to reduce senescent cell burden prior to vaccinating and boosting with flu nucleoprotein (NP) (Figure 1A). Dasatinib and Quercetin (D + Q) combination treatment is one of the most well‐characterized senolytics and have been shown to eliminate senescent cells. Dasatinib (D) inhibits several kinases including SRC and ABL and is currently used as a second line chemotherapeutic agent against imatinib‐resistant chronic myeloid leukemia (Kantarjian et al. 2006; Talpaz et al. 2006). Quercetin (Q) is a naturally occurring flavonoid and serves as a nonspecific kinase inhibitor, targeting multiple pathways and molecules including PI3K/AKT, BCL‐2, and others (Malavolta et al. 2016; Reyes‐Farias and Carrasco‐Pozo 2019). In combination, D + Q has enhanced senolytic activity compared to either agent alone, selectively eliminating a broader range of senescent cell types (Zhu et al. 2015).

Surprisingly, D + Q had no significant impact on serum NP IgG antibody titers or total number or frequency of NP‐specific CD4 T cells in the spleen 30 days post initial vaccination in aged mice (Figure 1B–D). Interestingly, there was a main treatment effect for D + Q for NP‐specific CD4 T cell frequency (Figure 1D). Although we did not see clear improvements in serum antibodies or NP‐specific T cells with D + Q in aged mice, we have previously shown that clearing senescence using the p16‐3MR transgenic mouse model resulted in altered T cell memory responses (Torrance et al. 2024). Thus, we next sought to evaluate further protective responses via sublethal flu challenge.

### 2.2 Effects of D + Q on Flu‐Induced Weight Loss Following Vaccination

While aging leads to humoral and cell‐mediated immune impairment, this is not always clearly indicated by antibody quantity. Thus, to evaluate the functionality of the immune memory generated from vaccination, young and aged mice were treated with D + Q, vaccinated and boosted with flu NP, then infected with a sublethal dose of A/PR/8/34 H1N1 influenza virus (PR8) (Figure 1A). As we and others have previously reported (Keilich et al. 2020), NP vaccination protected young mice completely from flu‐induced weight loss compared to unvaccinated, treatment‐matched counterparts (Figure 1E). Similarly, aged mice were significantly protected from flu‐induced weight loss compared to unvaccinated, treatment‐matched counterparts, albeit to a lesser degree than young mice (Figure 1F). D + Q treatment only transiently improved flu‐induced weight loss at 15 days post infection (DPI) in unvaccinated aged mice compared to vehicle‐treated unvaccinated aged counterparts (Figure 1F).

### 2.3 Effects of D + Q on CD4 T Cell Responses and Antibody Responses to Influenza Infection Following Vaccination

We proceeded to evaluate CD4 T cell responses in the draining mediastinal lymph node (MLN, Figures S1 and S2) and lungs (Figures S3 and S4) during subsequent sublethal flu challenge via flow cytometry (gating strategy in Figure S7). In the MLN, D + Q treated vaccinated aged mice had fewer NP‐specific CD4 T cells compared to vehicle counterparts at 9 DPI, however this is reversed with greater NP‐specific CD4 T cells numbers in the MLN at 12 DPI (Figure S2B,C). D + Q treated vaccinated aged mice showed altered kinetics in some NP‐specific CD4 T cell subsets, including a delay in peak numbers of NP‐specific Tregs, Tfh, T helper 2 cells (Th2), and Tfr in the MLN (Figure S2). However, these changes were of small magnitude, and this pattern was not seen in cell frequencies (Figure S1). Despite these changes in dynamics of NP‐specific CD4 T cell responses in the MLN with D + Q, these changes were not reflected in the lungs (Figures S3 and S4). In fact, there were no differences in NP‐specific CD4 T cell frequency or number in the lungs at 7, 9, or 12 DPI (Figure S3A–C and Figure 4A–C). Further, the only notable change in lungs was a decrease in NP‐specific Treg numbers at 7 DPI in D + Q treated vaccinated mice (Figure S4M).

Interestingly, unvaccinated aged mice only had trending increased NP‐specific Th2 and NP‐specific Tfh cell numbers and no changes in Tregs at 7 DPI in the MLN (Figure S2G,J,M), contradicting our previously reported CD4 T cell changes during primary flu infection with D + Q treatment. Unexpectedly, D + Q treated unvaccinated young mice had trending increased NP‐specific CD4 T cell numbers at 9 DPI in the MLN, followed by a significant decrease at 12 DPI (Figure S2B,C). These mice also had a significant increase in NP‐specific Th1 numbers and a significant decrease in NP‐specific Treg numbers in the MLN at 12 DPI (Figure S2F,O).

Despite the changes to NP‐specific Tfh responses observed in D + Q treated vaccinated aged mice, no effects were seen in serum or BAL NP and total PR8 (whole virus) IgG antibody titers at 9, 12, or 15 DPI in this group (Figure S5). Some differences in antibody responses were observed in unvaccinated aged mice, including a transient increase in serum NP IgG antibodies in the D + Q treated group compared to vehicle counterparts at 12 DPI and a decrease in serum total PR8 IgG antibody titers at 15 DPI (Figure S5B,F). Additionally, young D + Q treated vaccinated mice had increased serum NP IgG antibodies at 20 DPI compared to vehicle counterparts (Figure S5H).

### 2.4 Effects of D + Q on CD8 T Cell Responses to Influenza Infection

**FIGURE 2:**
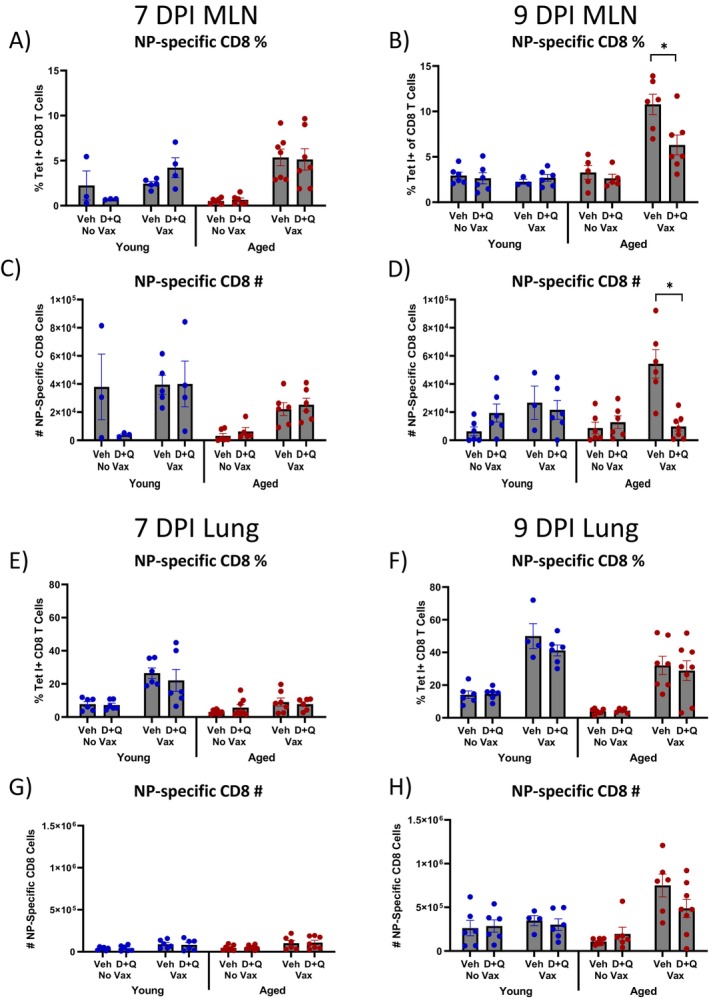
D + Q treatment prior to vaccination decreases CD8 T cell responses in the mediastinal lymph node (MLN) at 9 days post infection (DPI) in aged mice. Young (3–5 months old) and aged (18–20 months old) C57BL/6JN (B6) mice were treated with senolytic combination dasatinib plus quercetin (D + Q) or vehicle control prior to being vaccinated and boosted with flu nucleoprotein (NP) with alum or PBS with alum control. Mice were subsequently infected with a sublethal dose of PR8 H1N1 influenza, and NP‐specific CD8 T cells were evaluated in the MLN and lungs by flow cytometry using flu NP MHC I tetramer staining. (A, B) Frequency of NP‐specific CD8 T cells was evaluated at 7 and 9 DPI, respectively, in the MLN. (C, D) Numbers of NP‐specific CD8 T cells were evaluated at 7 and 9 DPI, respectively, in the MLN. (E, F) Frequency of NP‐specific CD8 T cells was evaluated at 7 and 9 DPI, respectively, in the lungs. (G, H) Numbers of NP‐specific CD8 T cells were evaluated at 7 and 9 DPI, respectively, in the lungs. Data are presented as mean ± standard error of the mean (SEM). Two‐way ANOVA was performed, followed by Šidák's test for multiple comparisons. Results were considered significant at *p* < 0.05. *N* = 3–8/group.

We next evaluated NP‐specific CD8 T cell responses in the MLN and lungs during subsequent sublethal flu challenge. Although there were no changes in NP‐specific CD8 T cell responses in the MLN at 7 DPI (Figure 2A,C), D + Q treated vaccinated aged mice had significantly lower NP‐specific CD8 T cell frequency and cell number in the MLN at 9 DPI compared to vehicle counterparts (Figure 2B,D). These changes were not observed in the lungs at 7 and 9 DPI (Figure 2E–H) and only occurred in aged mice. Thus, we next further interrogated flu‐specific CD8 T cell populations in the MLN and lungs.

**FIGURE 3:**
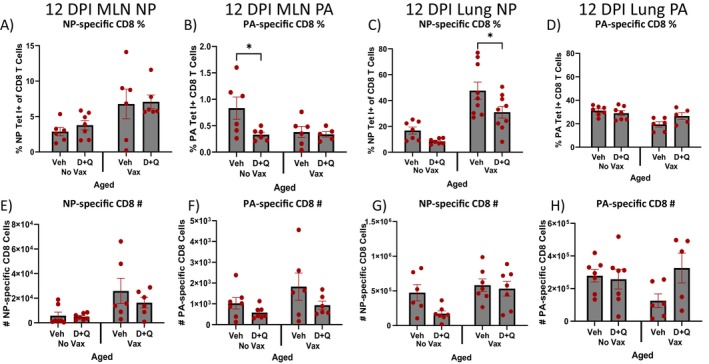
D + Q treatment prior to vaccination alters CD8 T cell immunodominance hierarchy in the lungs at 12 days post infection (DPI) in the lungs of aged mice. Young (3–5 months old) and aged (18–20 months old) C57BL/6JN (B6) mice were treated with senolytic combination dasatinib plus quercetin (D + Q) or vehicle control prior to being vaccinated and boosted with flu nucleoprotein (NP) with alum or PBS with alum control. Mice were subsequently infected with a sublethal dose of PR8 H1N1 influenza, and NP‐specific CD8 T cells were evaluated in the mediastinal lymph node (MLN) and lungs by flow cytometry using flu NP MHC I tetramer staining and flu acidic polymerase (PA) MHC I tetramer staining. (A, B) Frequency of NP‐specific and PA‐specific CD8 T cells was evaluated at 12 DPI in the MLN of aged mice. (C, D) Frequency of NP‐specific and PA‐specific CD8 T cells was evaluated at 12 DPI in the lungs of aged mice. (E, F) Numbers of NP‐specific and PA‐specific CD8 T cells were evaluated at 12 DPI in the MLN of aged mice. (G, H) Numbers of NP‐specific and PA‐specific CD8 T cells were evaluated at 12 DPI in the lungs of aged mice. Data are presented as mean ± standard error of the mean (SEM). Two‐way ANOVA was performed, followed by Šidák's test for multiple comparisons. Results were considered significant at *p* < 0.05. *N* = 5–9/group.

NP‐specific CD8 T cell frequency and number were not different in the MLN of D + Q treated vaccinated aged mice at 12 DPI (Figure 3A,E). However, these mice had significantly lower NP‐specific CD8 T cell frequency, but not cell number in the lungs at 12 DPI (Figure 3C,G). Simultaneously, a trending increase in CD8 T cell frequency and number specific for flu acidic polymerase (PA‐specific), the other known immunodominant flu antigen (Yager et al. 2008), was observed with D + Q treatment in vaccinated aged mice compared to vehicle counterparts (Figure 3D,H). It has been shown that during primary flu infection immunodominance is shared between NP and PA‐specific CD8 T cells, while secondary responses are dominated by NP‐specific T cells (La Gruta et al. 2006). Since we vaccinated with NP, we anticipated a primarily NP‐specific CD8 T cell response during flu challenge, as this would be a secondary response. However, D + Q treated vaccinated aged mice did not follow this expected immunodominance hierarchy.

D + Q treated unvaccinated aged mice showed a decrease in PA‐specific CD8 T cell frequency, but not cell number in the MLN at 12 DPI (Figure 3B,F). Importantly, D + Q treated unvaccinated aged mice also had a trending decrease in NP‐specific CD8 T cell numbers, but not frequency in the lungs at 12 DPI (Figure 3C,G), matching our previous findings following elimination of p16 expressing senescent cells in p16‐3MR mice (Torrance et al. 2024). Collectively, these results suggest a strong impact of D + Q treatment on lung NP‐specific CD8 T cell responses in aged mice, regardless of vaccination status.

### 2.5 Effects of Senolytics on Lung Viral Load and Influenza Outcomes

**FIGURE 4:**
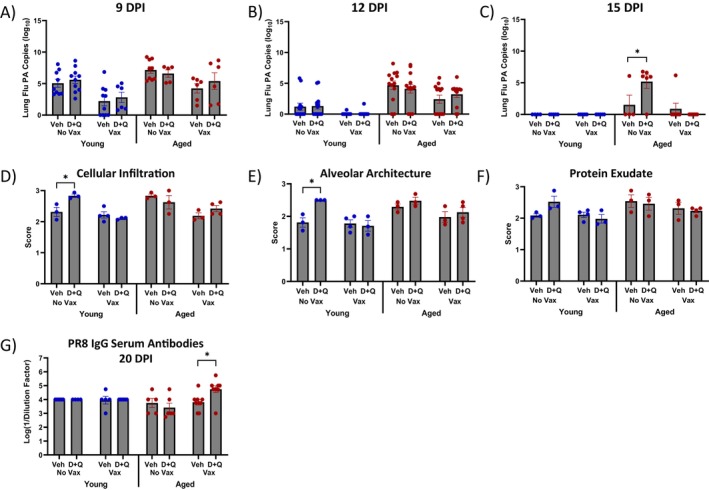
D + Q treatment prior to vaccination does not impact viral load or lung pathology in vaccinated aged mice but increases serum PR8 IgG antibody titers at 20 days post infection (DPI). (A–C) Viral replication was quantified in the lungs at various DPI by RT‐qPCR for flu acid polymerase (PA) gene copies. (D–F) Lung pathology was evaluated, and using H&E staining, scores were generated by 4 blinded evaluators. (G) TPR8 IgG serum antibodies were examined by ELISA from serum collected via cardiac puncture. Data are presented as mean ± standard error of the mean (SEM). Two‐way ANOVA was performed, followed by Šidák's test for multiple comparisons. Results were considered significant at *p* < 0.05. *N* = 7–9/group for flow cytometry experiments. *N* = 3–4/group for histology experiments.

To determine if changes in flu‐specific CD8 T cell immunodominance hierarchy impacted flu outcomes, we next assessed lung viral load and lung pathology. As expected and previously reported (Keilich et al. 2020), young and aged vaccinated mice had lower viral load compared to unvaccinated counterparts at 9 and 12 DPI, respectively (Figure 4A,B). Further, there were no differences in lung viral load at 15 DPI in D + Q treated vaccinated aged mice (Figure 4C). Surprisingly, unvaccinated D + Q treated aged mice had significantly higher lung viral load compared to vehicle counterparts at 15 DPI (Figure 4C), which may be partly due to the trending decreased NP‐specific CD8 T cell numbers at 12 DPI (Figure 3G).

No significant changes in lung pathology, including cellular infiltration, alveolar architecture and protein exudate, were observed in aged mice, regardless of D + Q treatment or vaccination at 20 DPI (Figure 4D–F, Figure S6). Unexpectedly, unvaccinated young mice treated with D + Q exhibited more severe lung pathology at 20 DPI (Figure 4D–F), evidenced by increased cell infiltration and worse alveolar architecture compared to their vehicle‐treated counterparts. It is possible that D + Q treatment may interfere with the key role of senescence during lung repair (Reyes et al. 2022). If these mechanisms are significantly disrupted in a persistent manner, it could explain the worsened lung pathology seen in D + Q treated, unvaccinated young mice. Further studies are needed to thoroughly investigate the long‐term effects of D + Q on tissues such as the lung, providing a clearer understanding of how D + Q treatment drives sustained impacts on lung repair well beyond its administration and clearance. Despite limited differences in CD4 T cell responses and vaccine‐induced (NP‐specific) antibodies, D + Q treated vaccinated aged mice had increased serum total PR8 (whole virus) IgG antibody titers at 20 DPI (Figure 4G). This is interesting given there were no persistent differences in serum or bronchioalveolar lavage fluid (BAL) NP or total PR8 (whole virus) IgG antibodies in D + Q treated vaccinated aged mice at other evaluated timepoints (Figure S5). This indicates that D + Q treatment may promote *de novo* antibody responses induced by infection or induce more broad, cross‐reactive antibody responses during infection.

## 3 Discussion

This study demonstrates that D + Q treatment prior to NP vaccination did not improve vaccine‐induced NP IgG antibody titers, CD4 T cell responses in the spleen or overall flu outcomes including flu‐induced weight loss, lung viral load and lung pathology. This was unexpected, given that other studies have reported benefits in immune responses with D + Q treatment in infections like COVID‐19, using models like the K18‐ACE2 transgenic mouse and human brain organoids (Aguado et al. 2023; Pastor‐Fernández et al. 2023). Similarly, D + Q, fisetin, and elimination of p16 expressing senescent cells in the *INK‐ATTAC* transgenic mouse model all showed a significant delay or reduction in β‐coronavirus related mortality in male and female mice with normal microbial exposure (Camell et al. 2021). While those studies showed improved overall infection outcomes, such as decreased inflammation and extended survival, our findings reveal more limited effects without stark improvements in flu outcomes. It is possible that the differences in infection types, for example, systemic vs. localized respiratory, could explain some of the divergent responses observed.

Despite not observing any significance in vaccine‐induced protection, like antibody titers and overall CD4 T cell responses in the spleen, we observed a main treatment effect of D + Q on NP‐specific CD4 T cells. Nevertheless, given the possibility that changes in vaccine efficacy with D + Q could manifest as functional protection rather than isolated immune readouts, we tested the effectiveness of both humoral and cell‐mediated protection by sublethal flu challenge with A/PR/8/34 (PR8) H1N1 influenza. As previously detailed by us and others, aged mice experience prolonged and more severe weight loss compared to their young counterparts during infection (Bartley et al. 2016; Keilich et al. 2023), while NP vaccination completely prevents flu‐induced weight loss in young mice and reduces flu‐induced weight loss in aged mice (Keilich et al. 2020).

Our study demonstrated an unexpected interaction between D + Q treatment and vaccination on CD8 T cell responses during subsequent infection in aged mice. D + Q treated vaccinated aged mice had a decrease in NP‐specific CD8 T cell frequency in the MLN at 9 DPI, followed by a similar sustained decrease in the lungs at 12 DPI. This decrease in NP‐specific CD8 T cell frequency in the lungs was accompanied by trending increases in PA‐specific CD8 T cell frequency and cell number in D + Q treated vaccinated aged mice. It is not clear if the decrease in NP‐specific CD8 T cells drives the expansion of PA‐specific CD8 T cells in a compensatory mechanism or if the expansion of PA‐specific CD8 T cells drives the reduction in NP‐specific CD8 T cell frequency. More research is necessary to understand the mechanism underlying these changes. Nonetheless, these findings suggest that D + Q impacts the immunodominance hierarchy of flu‐specific CD8 T cells in vaccinated aged mice. It has previously been reported that early and high level of expression of NP, either through direct or cross presentation, plays a crucial role in determining response magnitude (La Gruta et al. 2006). Also, the density of epitopes on the surface of individual antigen‐presenting cells (APCs) may be the key factor in determining immunodominance hierarchy. This suggests that in our model, D + Q may impact direct or cross presentation and/or epitope density of NP on APCs, resulting in our diminished NP‐specific CD8 T cell recall response in the MLN and lungs during flu infection. It is also possible that D + Q may be impacting signaling pathways that drive NP‐specific and PA‐specific CD8 T cell responses. For example, D + Q may impact CD40 signaling through its reduction of NF‐kB pathway activity (Zhao et al. 2025). Since CD40 signaling is critical for NP‐specific CD8 T cell responses, its disruption could impair these responses. This would also provide an explanation for the declines in NP‐specific CD8 T cell responses in unvaccinated aged mice. It was surprising, however, that despite these notable differences to the immunodominance hierarchy, there were no impacts on overall flu outcomes, such as lung viral load or lung pathology, in our D + Q treated vaccinated aged mice.

Although the primary aim of this study was to investigate the impact of D + Q treatment on vaccination responses, we observed striking changes in unvaccinated aged mice. D + Q treated unvaccinated aged mice had significantly lower PA‐specific CD8 T cell frequency in the MLN and a trending decrease in NP‐specific CD8 T cells in the lungs at 12 DPI compared to vehicle treated counterparts. Importantly, unvaccinated aged mice treated with D + Q also showed impaired viral clearance and lower serum total PR8 (whole virus) IgG antibody titers at 15 DPI. We previously showed that D + Q treatment prior to primary flu infection resulted in a transient decrease in NP‐specific CD8 T cells at 10 days post infection without significantly impacting lung viral load (Torrance et al. 2023). While our previous study showed that D + Q had no effect on overall primary flu infection outcomes, our current study reports impairment in viral clearance. In our previous study, primary PR8 infection occurred 5 days after the last dose of D + Q, while in our current studies, PR8 infection occurred 35 days after the last dose of D + Q. These findings suggest disparate effects of D + Q treatment on viral clearance depending on administration timeline and proximity to infection potentially pointing to long‐term disadvantages. Nonetheless, our findings of decreased immune cell infiltration in the lungs during infection were consistent with previous findings in our lab with clearance of p16‐expressing senescent cells with transgenic p16‐3MR mice (Torrance et al. 2024). Others have also reported that pretreatment with senolytics, including D + Q and fisetin, led to decreased cell infiltration into the lungs of aged female mice during similar sublethal PR8 infection; however, conversely to our findings in male mice, these studies did not report impaired lung viral clearance (Luna et al. 2025). Additional studies are required to unveil the mechanisms driving altered anti‐viral responses with D + Q in aging, as well as these notable sex differences in lung viral load. This is particularly important as it has been shown that senolytics have differential effects in males compared to females in various measures, including SASP levels, energy metabolism markers, body weight, and behavior (Fang et al. 2023; Garbarino et al. 2020).

Following flu infection in D + Q‐treated vaccinated aged mice, an interesting pattern was observed with lower cell numbers in various CD4 T cell subsets (Tregs, Tfh, Th2, and Tfr) at 9 DPI, followed by an increase at 12 DPI compared to vehicle treated counterparts. These changes were small in magnitude, and this pattern was not seen in cell frequencies. Further, there was no evidence of negative impacts of these changes, such as reduced support for B cells evidenced by serum antibody changes. It is likely that the small magnitude of cell number changes, although statistically significant, may not be biologically meaningful. We previously showed that D + Q shifts CD4 T cell differentiation away from Tregs and towards Th2s during primary infection (Lorenzo et al. 2022). In our study, we observed decreased Tregs cell number at various time points in the MLN and lungs with D + Q treatment in aged mice, though not sustained. We did not observe increased Th2s, however, in our study. This suggests that D + Q may ameliorate known age‐related increases in Treg populations that occur early in influenza infection (Williams‐Bey et al. 2011), however, sustained suppression may be dependent upon shifts in other CD4 T cell subsets. It is possible these changes are due to differences in experimental timelines between the previous study and this study, as previously discussed.

Tfr CD4 T cells, characterized by the co‐expression of FoxP3 and Bcl6 and suppression of antibody responses, are known to increase with age (Sage et al. 2015). While NP‐specific Tfr cells in D + Q treated vaccinated mice in the MLN at 12 DPI were increased, this did not negatively impact antibody responses and thus may not be biologically meaningful. Overall, the alterations in CD4 T cell responses in D + Q treated vaccinated mice groups were small in magnitude and did not seem to have a biological impact that we observed.

Although the primary aim of this study was to investigate the potential benefits of D + Q treatment on the immune response to vaccination, we also observed some intriguing changes in the unvaccinated control groups. Aged D + Q treated unvaccinated mice had trending increased numbers of NP‐specific Th2 and NP‐specific Tfh cells, as well as decreased NP‐specific Tfr frequency in the MLN and lungs at 7 DPI. This corresponded with increased serum NP IgG antibody titers at 12 DPI; however, at 15 DPI, this group had significantly lower serum total PR8 IgG antibody titers. Importantly, aged unvaccinated D + Q treated mice had impaired viral clearance at 15 DPI, suggesting that improvements in CD4 T cell differentiation did not have positive effects on overall flu outcomes.

We did not expect D + Q treatment to have a significant impact on young mice due to their known low senescence burden; however, we observed increased lung pathology in D + Q treated unvaccinated young mice. This was surprising as we did not observe any deficits in lung viral clearance or antibody titers in these groups. Interestingly, others have shown that p16‐expressing cells are integral to lung repair and their elimination negatively impacts lung pathology (Reyes et al. 2022). It is possible that flu infection further exacerbates this negative impact; however, why only young, unvaccinated mice treated with D + Q have worse pathology is unclear. We did note, however, significantly more NP‐specific T helper 1 (Th1) cells and significantly fewer NP‐specific Tregs in the MLN at 12 DPI in D + Q treated unvaccinated young mice compared to vehicle counterparts. It is possible that these changes, alongside the known role of p16‐expressing cells in lung repair (Reyes et al. 2022), negatively impact the resolution of flu in D + Q treated unvaccinated young mice. Additional studies are required to understand how D and Q, which have short half‐lives and are cleared quickly, can alter lung repair long after administration. These findings underline the importance of future mechanistic studies on the impact of senolytics in different ages, tissues, and diseases.

Interestingly, we did not observe age‐related differences in measures including cell infiltration, lung architecture, or antibody responses with D + Q or vaccination, though it's important to note that histology was only examined at 20 DPI. Additionally, it is well known that antibody quantities do not always change with aging, but rather impaired humoral responses are also indicated by impaired antibody quality (Henry et al. 2019). The unexpected findings of impaired viral clearance in unvaccinated aged mice alongside negative impacts on young lung histology highlight the need for further research exploring adjustments in D + Q treatment dosage or scheduling to examine potential differences in acute and chronic impacts. Additionally, these studies would be valuable to better define the optimal regimen for enhancing vaccine and infection responses in aging models.

While our studies did not show stark improvement in flu vaccine and infection responses, D + Q treatment modulated CD8 T cell immunodominance during subsequent flu challenge. NP‐specific CD8 T cell frequency was decreased, accompanied by increased PA‐specific CD8 T cell frequency and numbers in the lungs at 12 DPI. Despite this modulation of CD8 T cell immunodominance hierarchy, D + Q treated vaccinated aged mice achieved similar flu outcomes in terms of lung viral load and lung pathology compared to vehicle counterparts. D + Q treated vaccinated aged mice also had higher serum total PR8 (whole virus) IgG antibody titers at 20 DPI, but no differences in serum or BAL total PR8 (whole virus) IgG or NP IgG antibody titers at any earlier timepoints. This suggests that D + Q may modulate humoral responses with potential influence on immunodominance, cross‐reactive protection, or de novo responses. These findings are novel as others have not shown these changes with senolytics in other infection models. It remains unclear if these findings are specific to PR8 flu infections and D + Q treatment, or if they are more widely generalizable to other pathogens and/or senolytic treatments. Further studies are required to understand the mechanisms underlying these changes in adaptive immunity following D + Q treatment and the potential effects of D + Q on other vaccination and infection responses. While our studies do not suggest that D + Q can improve flu vaccination responses, the modulation of T cell immunodominance and increased PR8 (whole flu) IgG antibody titers is an important finding that can potentially be harnessed to modulate immune responses once mechanisms are understood more thoroughly.

## 4 Methods

### 4.1 Mice

All experiments used young (3–5 months) and aged (18–20 months) C57BL/6JN male mice provided by the National Institute on Aging Rodent Colony or purchased from Jackson Laboratories. All mice were housed at UConn Health in a climate‐controlled environment with a 12‐h light/dark cycle and fed a standard chow diet and water ad lib. All mice underwent examination at the time of sacrifice, and animals with visible pathologies (tumors, etc.) were excluded. Mice were cared for in accordance with the recommendations in the Guide for the Care and Use of Laboratory Animals of the National Institutes of Health. All procedures performed were approved by the UConn Health Institutional Animal Care and Use Committee (IACUC).

### 4.2 Senolytic Treatment

Mice were treated with a combination of 5 mg/kg/day dasatinib (D) and 50 mg/kg/day quercetin (Q) or an equal volume of vehicle control consisting of 10% ethanol, 30% polyethylene glycol, and 60% Phosal 50PG via oral gavage. As shown in Figure 1A, mice were treated for 3 days consecutively, allowed to rest for 1 week, then treated for a second round for another 3 days before resting for 5 days prior to vaccination. Rest periods ensured sufficient time for clearance of D and Q prior to vaccination, given their half‐lives of 4 and 11 h, respectively (Christopher et al. 2008; Graefe et al. 2001). This treatment schedule has been widely used for administering D + Q and has been shown to reduce senescent burden, increase lifespan, and alleviate aspects of age‐related dysfunction (Xu et al. 2018).

### 4.3 Vaccination

Recombinant A/PR/8/ influenza nucleoprotein (NP) was generated by the Protein Expression Core at UConn Health. Vaccine was prepared by combining NP protein and Imject Alum (Thermo Scientific) at a 1:1 ratio and was administered intraperitoneally at a final concentration of 30 μg NP in 100 μL per mouse. Control mice were administered PBS and Imject Alum (Thermo Scientific) at a 1:1 ratio in 100 μL per mouse. Mice received the first dose of vaccination 5 days after the last D + Q treatment and a second vaccination (boost) 10 days after initial vaccination, as shown in Figure 1A.

### 4.4 Viral Infection

Mice were anesthetized with isoflurane and intranasally infected with 50 μL of 500 EID_50_ of A/PR/8/34 (PR8) influenza virus. Mice were weighed daily to evaluate infection progression.

### 4.5 Viral Load Quantification

Following euthanasia, lungs were flash frozen in liquid nitrogen. Lung tissue was homogenized, and RNA was isolated using a standard trizol/chloroform (Invitrogen Life Technologies and Sigma Aldrich, respectively) extraction per the manufacturer's protocol. cDNA was synthesized using the iScript cDNA Synthesis kit (Bio‐Rad) per the manufacturer's protocol. Viral load was quantified by RT‐qPCR for PR8 acid polymerase (PA) gene compared to a standard curve of known PA copy numbers as previously published and validated (Jelley‐Gibbs et al. 2007). The primer and probe sequences utilized were forward primer, 5′‐CGGTCCAAATTCCTGCTGA‐3′ and reverse primer, 5′: CATTGGGTTCCTTCCATCCA‐3′ probe, 5′‐6‐FAMCCAAGTCATGAAGGAGAGGGAATACCGCT‐3′ (Integrated DNA Technologies).

### 4.6 Antibody Quantification

Bronchoalveolar lavage (BAL) was collected at the time of sacrifice by flushing the lungs with 1 mL of PBS. Supernatant was collected after centrifugation to separate cells and debris. Serum was collected via blood from cardiac puncture immediately postmortem. 96‐well plates were coated with either whole PR8 virus or recombinant flu nucleoprotein (NP). BAL and serum samples were serially diluted 10‐fold and transferred to previously coated plates in duplicate. IgG antibody levels were quantified via horseradish peroxidase‐conjugated anti‐IgG antibody (Southern Biotech). Titer was determined as the highest dilution with a signal over the background (mean plus 2 standard deviations).

### 4.7 Tissue Processing for Flow Cytometry

Following euthanasia, lungs were harvested and then mechanically and enzymatically digested (100 U/mL collagenase (Gibco)) in RPMI media containing 5% heat‐inactivated fetal bovine serum. Red blood cells were lysed using ACK lysis buffer (Gibco). Spleens were mechanically digested through 70 μm filters; then red blood cells were lysed using ACK lysis buffer (Gibco). Mediastinal lymph nodes were mechanically digested through 70 um filters. Cell counts were obtained from single cell suspensions using the LUNA‐FX7 Automated Cell Counter.

Single cell suspensions were stained for flow cytometry by incubating with Fc block (anti CD‐16/32, Thermo Fisher), then stained with NP311‐325 IAb MHC Class II tetramer, NP366‐374 H‐2Db MHC Class I tetramer or PA224‐233 H2‐Db MHC Class I tetramer (generated by the NIH Tetramer Core Facility). Cells were then stained with surface antibodies and then fixed with 1% paraformaldehyde or permeabilized using a FoxP3/Transcription factor fixation/permeabilization kit (Thermo Fisher). Samples that underwent permeabilization were stained with intracellular antibodies. Additional information about antibodies used can be found in Table S1. Data were acquired on Becton Dickinson (BD) LSR II and BD FACSymphony A5 SE Cell Analyzer and analyzed using FlowJo (BD) with fluorescence minus one (FMO) controls. The gating strategy used can be found in Figure S7.

### 4.8 Lung Histology

After sacrificing mice, lungs were immersed in 4% formalin for 24 h prior to transfer into 70% EtOH. Lungs were then sectioned at 5 μm thickness and stained by hematoxylin and eosin staining (H&E). Lungs were imaged at 10× without Z stacking using ZEISS Axioscan 7. H&E images were evaluated by 4 blinded evaluators with predetermined rubrics.

### 4.9 Statistics

Data are presented as mean ± standard error of the mean (SEM). Outliers were identified by mean ± 2 standard deviations. Statistical differences were evaluated by Two‐Way Anova with Sidak Corrections for Multiple Comparisons using GraphPad Prism10 software. *p* value of < 0.05, indicated by *, was considered significant.

## Author Contributions

J.M.B. conceived the experiments and secured funding. J.M.B. and A.N.C. designed experiments. A.N.C. performed all experiments with assistance from B.L.T., S.A.F., D.A.D., Z.H., D.E.T., N.M., K.A.G., and E.C.L. J.M.B. and A.N.C. analyzed and interpreted data with assistance from L.H. A.N.C. wrote the manuscript with critical feedback from all authors. All authors read and approved the final manuscript.

## Funding

This work was supported by the National Institute of Allergy and Infectious Diseases (R01AI173305), National Institute of Arthritis and Musculoskeletal and Skin Diseases (T32AR079114); American Federation for Aging Research; National Institute on Aging (P30AG067988).

## Conflicts of Interest

The authors declare no conflicts of interest.

## Supporting information


**Figure S1:** D + Q treatment prior to vaccination has no significant effects on frequency of NP‐specific CD4 T cell responses in the mediastinal lymph node during infection in aged mice. Young (3–5 months old) and aged (18–20 months old) C57BL/6JN (B6) mice were treated as in Figure 1A. (A–R) Frequency of total NP‐specific CD4 T cells and various NP‐specific CD4 T cell subsets were evaluated in the MLN by flow cytometry via flu NP MHC II tetramer staining. Data are presented as mean ± standard error of the mean (SEM). Two‐way ANOVA was performed, followed by Šidák's test for multiple comparisons. Results were considered significant at *p* < 0.05. *N* = 3–9/group.

**Figure S2:** D + Q treatment prior to vaccination has no significant effects on numbers of NP‐specific CD4 T cell responses in the mediastinal lymph node during infection in aged mice. Young (3–5 months old) and aged (18–20 months old) C57BL/6JN (B6) mice were treated as in Figure 1A. (A–R) Number of total NP‐specific CD4 T cells and various NP‐specific CD4 T cell subsets were evaluated in the MLN by flow cytometry via flu NP MHC II tetramer staining. Data are presented as mean ± standard error of the mean (SEM). Two‐way ANOVA was performed, followed by Šidák's test for multiple comparisons. Results were considered significant at *p* < 0.05. *N* = 3–9/group.

**Figure S3:** D + Q treatment prior to vaccination has minimal impacts on frequency of NP‐specific CD4 T cell responses in the lungs during infection in aged mice. Young (3–5 months old) and aged (18–20 months old) C57BL/6JN (B6) mice were treated as in Figure 1A. (A–R) Frequency of total NP‐specific CD4 T cells and various NP‐specific CD4 T cell subsets were evaluated in the lungs by flow cytometry via flu NP MHC II tetramer staining. Data are presented as mean ± standard error of the mean (SEM). Two‐way ANOVA was performed, followed by Šidák's test for multiple comparisons. Results were considered significant at *p* < 0.05. *N* = 3–8/group.

**Figure S4:** D + Q treatment prior to vaccination has minimal impacts on numbers of NP‐specific CD4 T cell responses in the lungs during infection in aged mice. Young (3–5 months old) and aged (18–20 months old) C57BL/6JN (B6) mice were treated as in Figure 1A. (A–R) Numbers of total NP‐specific CD4 T cells and various NP‐specific CD4 T cell subsets were evaluated in the lungs by flow cytometry via flu NP MHC II tetramer staining. Data are presented as mean ± standard error of the mean (SEM). Two‐way ANOVA was performed, followed by Šidák's test for multiple comparisons. Results were considered significant at *p* < 0.05. *N* = 4–9/group.

**Figure S5:** D + Q treatment prior to vaccination has no impact on NP or total PR8 (whole virus) IgG antibody titers in bronchoalveolar lavage or serum in aged mice up to 15 DPI. Young (3–5 months old) and aged (18–20 months old) C57BL/6JN (B6) mice were treated as in Figure 1A. (A–H) NP and PR8 (whole virus particle) IgG antibodies were quantified via ELISA at various days post‐infection in bronchoalveolar lavage (BAL) and serum. Data are presented as mean ± standard error of the mean (SEM). Two‐way ANOVA was performed, followed by Šidák's test for multiple comparisons. Results were considered significant at *p* < 0.05. *N* = 3–10/group.

**Figure S6:** Representative lung histology at 20 days post infection (DPI). Young (3–5 months old) and aged (18–20 months old) C57BL/6JN (B6) mice were treated as in Figure 1A. (A) Representative images of lungs were generated from 5 μm lung sections and stained by hematoxylin and eosin staining (H&E). Data are presented as mean ± standard error of the mean (SEM). Two‐way ANOVA was performed, followed by Šidák's test for multiple comparisons. Results were considered significant at *p* < 0.05.

**Figure S7:** Flow cytometric analysis of T cell phenotypes following flu infection. All T cell phenotyping utilized the gating strategy shown. All cells were gated on Lymphocytes (FSC‐A X SCA‐A), Single Cells (FCS‐H X FCS‐A), Live (Succinimidyl Ester‐A X FCS‐A) and CD4 (CD4+ X CD8‐) and CD8 (CD4‐ X CD8+) T cells. After, within the CD8 T cell population, NP‐specific CD8 T cells were gated using NP366‐374 H‐2Db MHC Class I tetramer (CD8+, NP MHC Class I tetramer+) and PA‐specific CD8 T cells were gated using PA 224–233 H2‐Db MHC Class I tetramer (CD8+, PA MHC Class I tetramer+). NP‐specific CD4 T cells were gated within the CD4 T cell population using NP311‐325 IAb MHC Class II tetramer (CD4+, NP MHC Class II tetramer+). NP‐Specific CD4 T cell subsets were gated within the NP‐Specific CD4 T cell population as follows: NP‐Specific T helper 1 cells (Th1: NP MHC Class II tetramer+, Tbet+), NP‐Specific T helper 2 cells (Th2: NP MHC Class II tetramer+, GATA3+), NP‐Specific T follicular helper cells (Tfh: NP MHC Class II tetramer+, Bcl6+/FoxP3‐), NP‐specific Regulatory T cells (Treg: NP MHC Class II tetramer+, Bcl6‐/FoxP3+), NP‐specific T follicular regulatory (Tfr: NP MHC Class II tetramer+, Bcl6+/FoxP3+).

**Table S1:** Extended antibody information for flow cytometry experiments.

## Data Availability

The data that support the findings of this study are available from the corresponding author upon reasonable request.
