# Supplementary figures and images for: Senolytic Treatment With Dasatinib and Quercetin Reshapes Influenza‐Specific CD8 T Cell Responses During Infection in Aged, Vaccinated Mice
Source: Aging Cell. 2025 Dec 29;25(1):e70345. doi: 10.1111/acel.70345 (PMC12748518; doi:10.1111/acel.70345)

Supplemental Figure 1

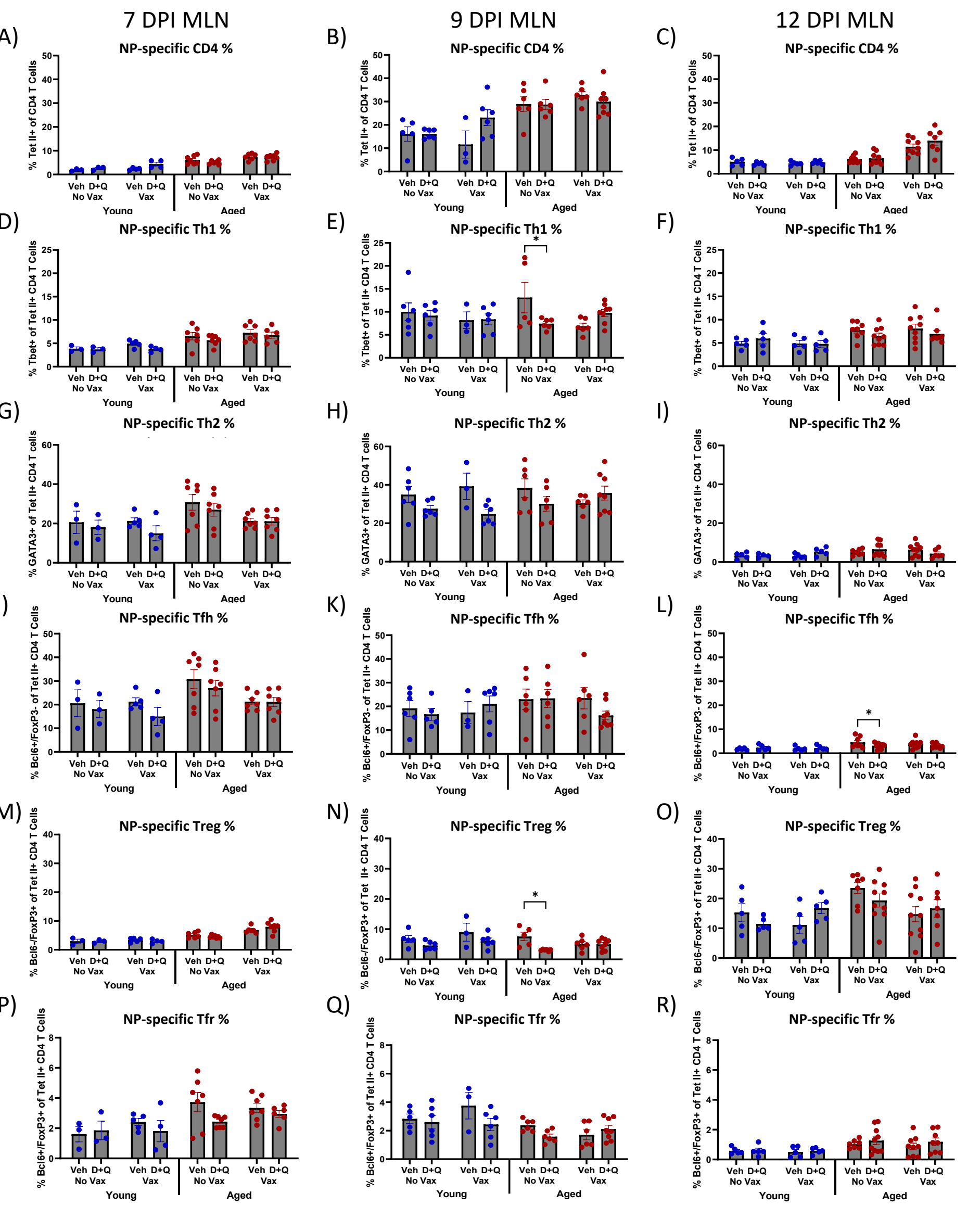

Supplement: Supplementary file 1 — Figure S1: D + Q treatment prior to vaccination has no significant effects on frequency of NP‐specific CD4 T cell responses in the mediastinal lymph node during infection in aged mice. Young (3–5 months old) and aged (18–20 months old) C57BL/6JN (B6) mice were treated as in Figure 1A. (A–R) Frequency of total NP‐specific CD4 T cells and various NP‐specific CD4 T cell subsets were evaluated in the MLN by flow cytometry via flu NP MHC II tetramer staining. Data are presented as mean ± standard error of the mean (SEM). Two‐way ANOVA was performed, followed by Šidák's test for multiple comparisons. Results were considered significant at p < 0.05. N = 3–9/group. [file ACEL-25-e70345-s003.pdf]

Supplemental Figure 2

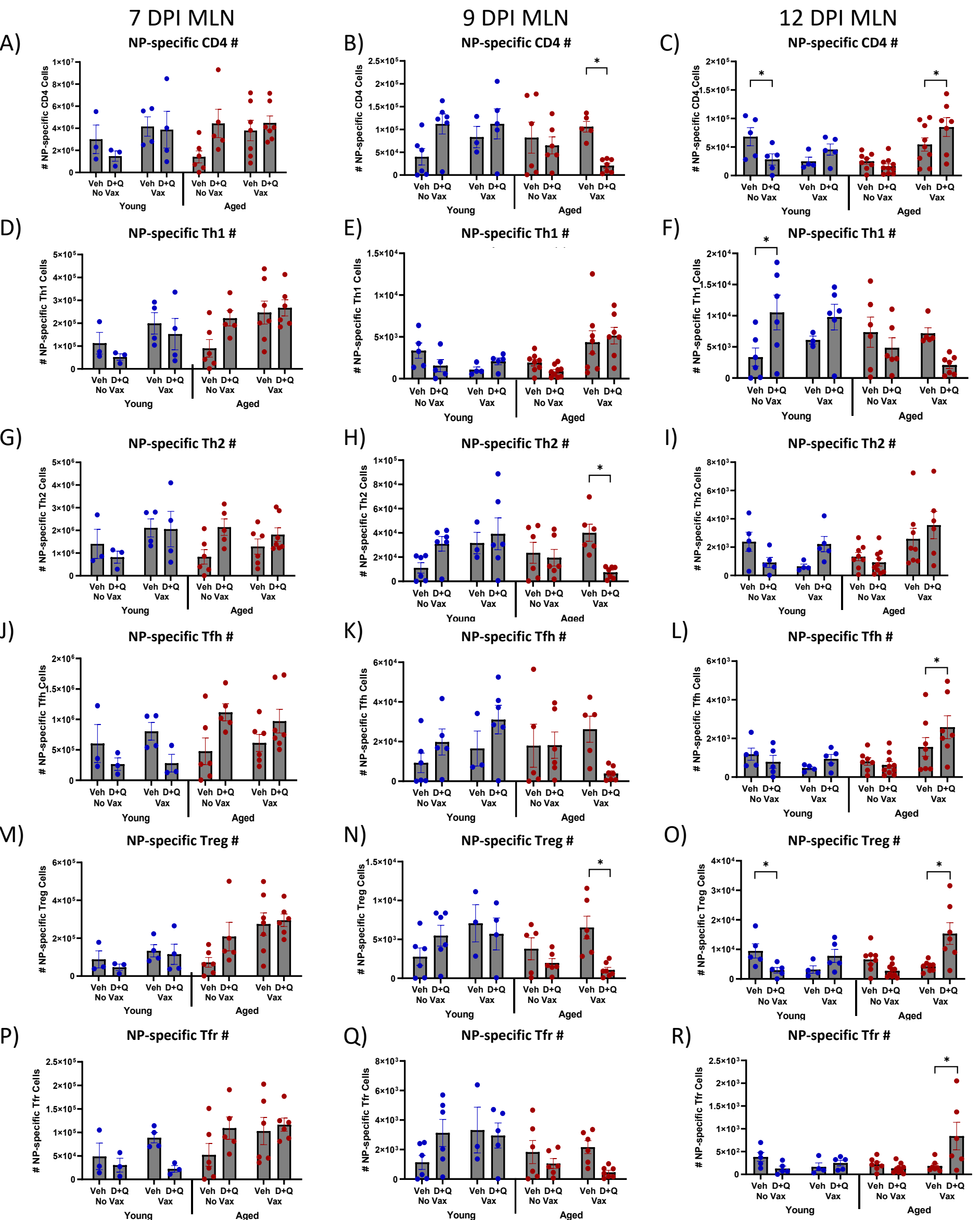

Supplement: Supplementary file 2 — Figure S2: D + Q treatment prior to vaccination has no significant effects on numbers of NP‐specific CD4 T cell responses in the mediastinal lymph node during infection in aged mice. Young (3–5 months old) and aged (18–20 months old) C57BL/6JN (B6) mice were treated as in Figure 1A. (A–R) Number of total NP‐specific CD4 T cells and various NP‐specific CD4 T cell subsets were evaluated in the MLN by flow cytometry via flu NP MHC II tetramer staining. Data are presented as mean ± standard error of the mean (SEM). Two‐way ANOVA was performed, followed by Šidák's test for multiple comparisons. Results were considered significant at p < 0.05. N = 3–9/group. [file ACEL-25-e70345-s004.pdf]

Supplemental Figure 3

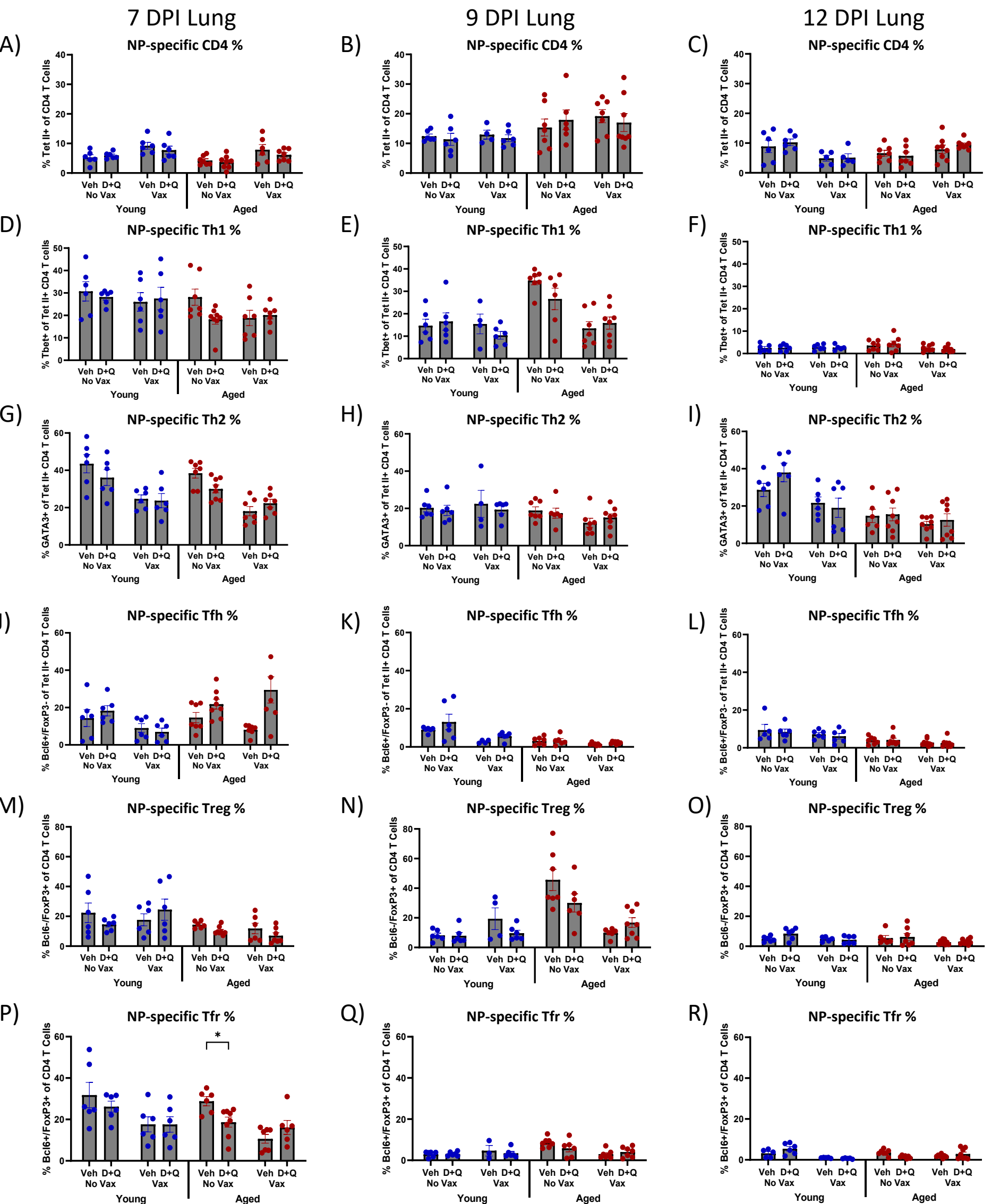

Supplement: Supplementary file 3 — Figure S3: D + Q treatment prior to vaccination has minimal impacts on frequency of NP‐specific CD4 T cell responses in the lungs during infection in aged mice. Young (3–5 months old) and aged (18–20 months old) C57BL/6JN (B6) mice were treated as in Figure 1A. (A–R) Frequency of total NP‐specific CD4 T cells and various NP‐specific CD4 T cell subsets were evaluated in the lungs by flow cytometry via flu NP MHC II tetramer staining. Data are presented as mean ± standard error of the mean (SEM). Two‐way ANOVA was performed, followed by Šidák's test for multiple comparisons. Results were considered significant at p < 0.05. N = 3–8/group. [file ACEL-25-e70345-s006.pdf]

Supplemental Figure 4

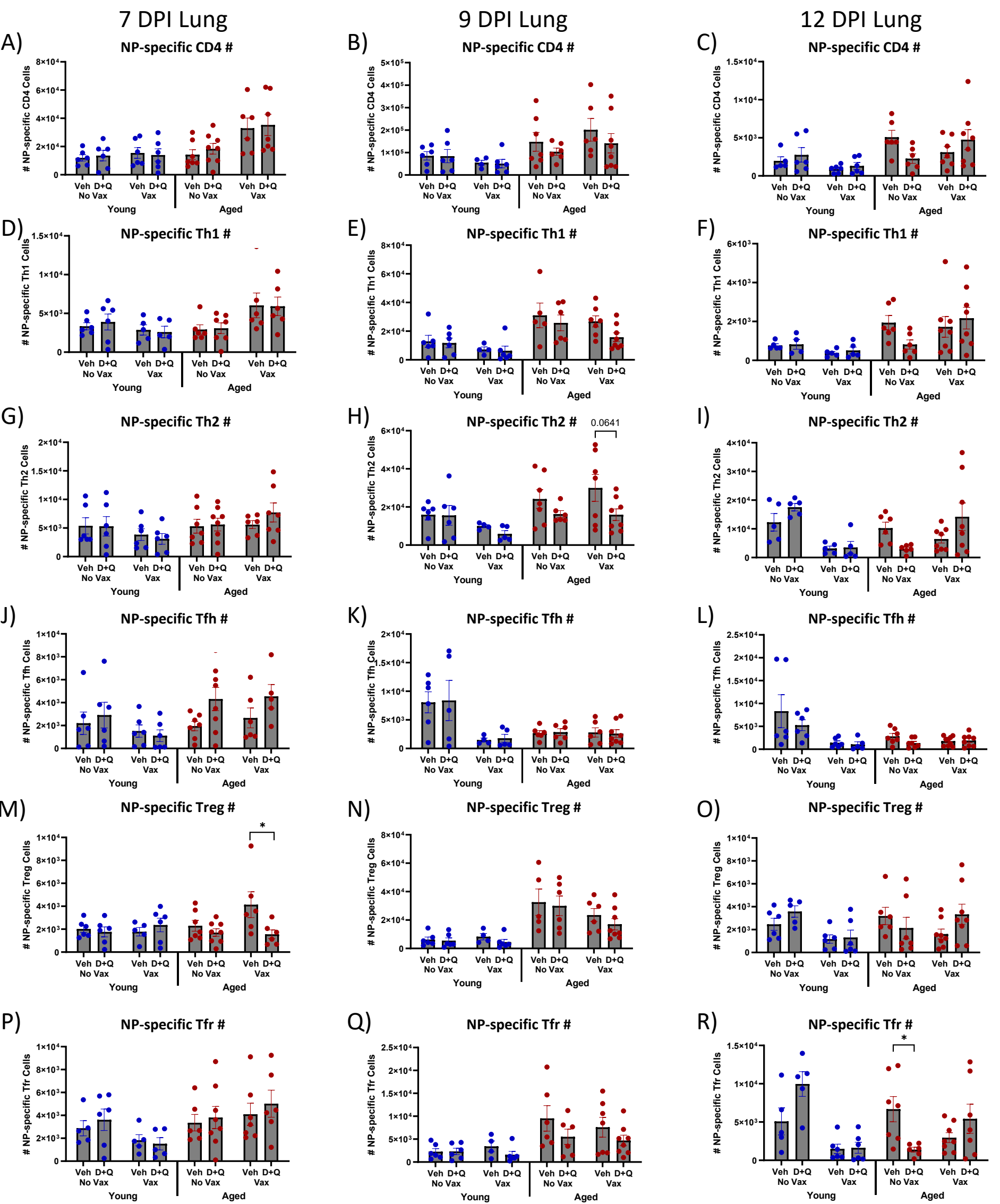

Supplement: Supplementary file 4 — Figure S4: D + Q treatment prior to vaccination has minimal impacts on numbers of NP‐specific CD4 T cell responses in the lungs during infection in aged mice. Young (3–5 months old) and aged (18–20 months old) C57BL/6JN (B6) mice were treated as in Figure 1A. (A–R) Numbers of total NP‐specific CD4 T cells and various NP‐specific CD4 T cell subsets were evaluated in the lungs by flow cytometry via flu NP MHC II tetramer staining. Data are presented as mean ± standard error of the mean (SEM). Two‐way ANOVA was performed, followed by Šidák's test for multiple comparisons. Results were considered significant at p < 0.05. N = 4–9/group. [file ACEL-25-e70345-s005.pdf]

Supplemental Figure 5

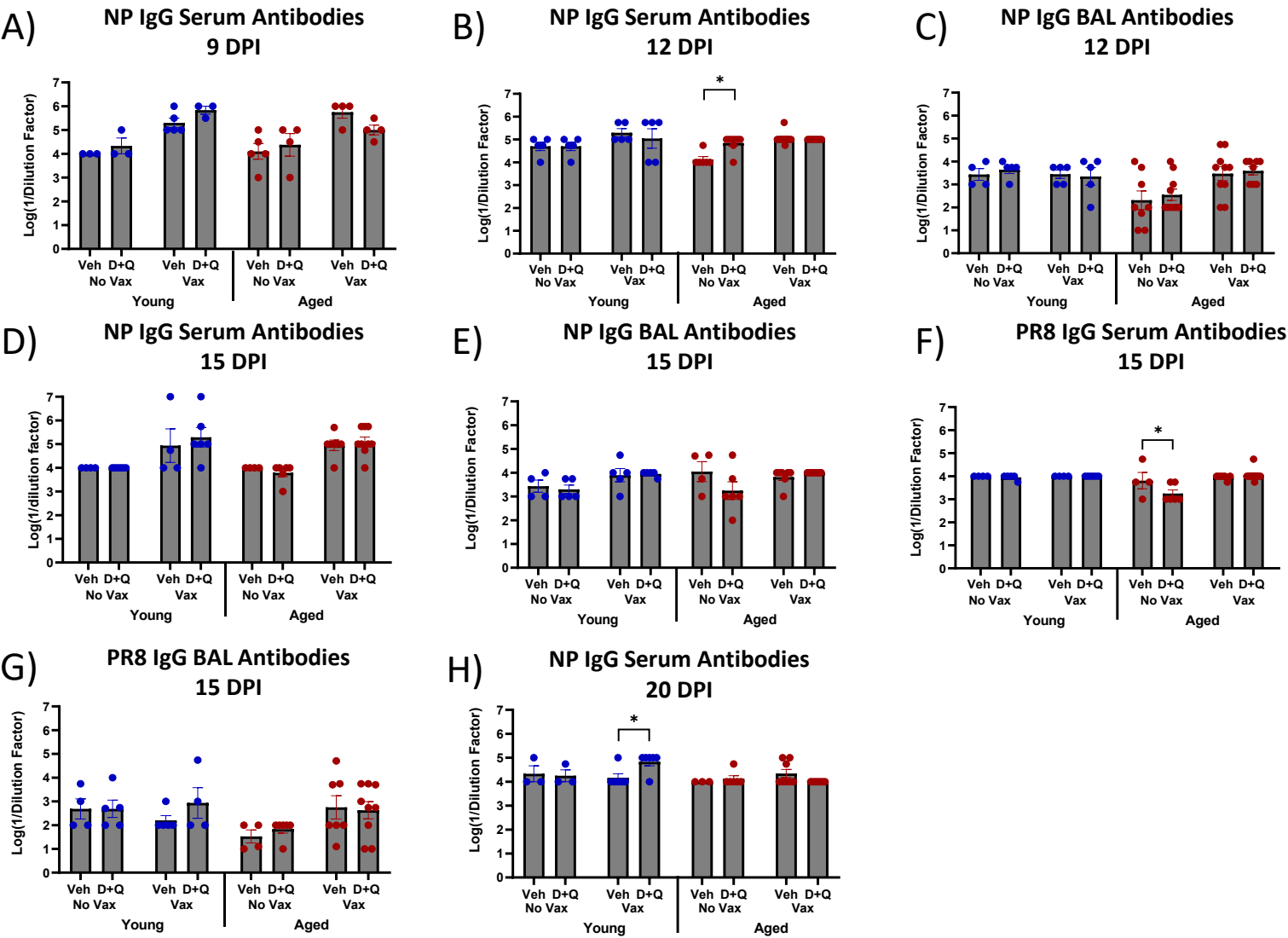

Supplement: Supplementary file 5 — Figure S5: D + Q treatment prior to vaccination has no impact on NP or total PR8 (whole virus) IgG antibody titers in bronchoalveolar lavage or serum in aged mice up to 15 DPI. Young (3–5 months old) and aged (18–20 months old) C57BL/6JN (B6) mice were treated as in Figure 1A. (A–H) NP and PR8 (whole virus particle) IgG antibodies were quantified via ELISA at various days post‐infection in bronchoalveolar lavage (BAL) and serum. Data are presented as mean ± standard error of the mean (SEM). Two‐way ANOVA was performed, followed by Šidák's test for multiple comparisons. Results were considered significant at p < 0.05. N = 3–10/group. [file ACEL-25-e70345-s002.pdf]

Supplemental Figure 6

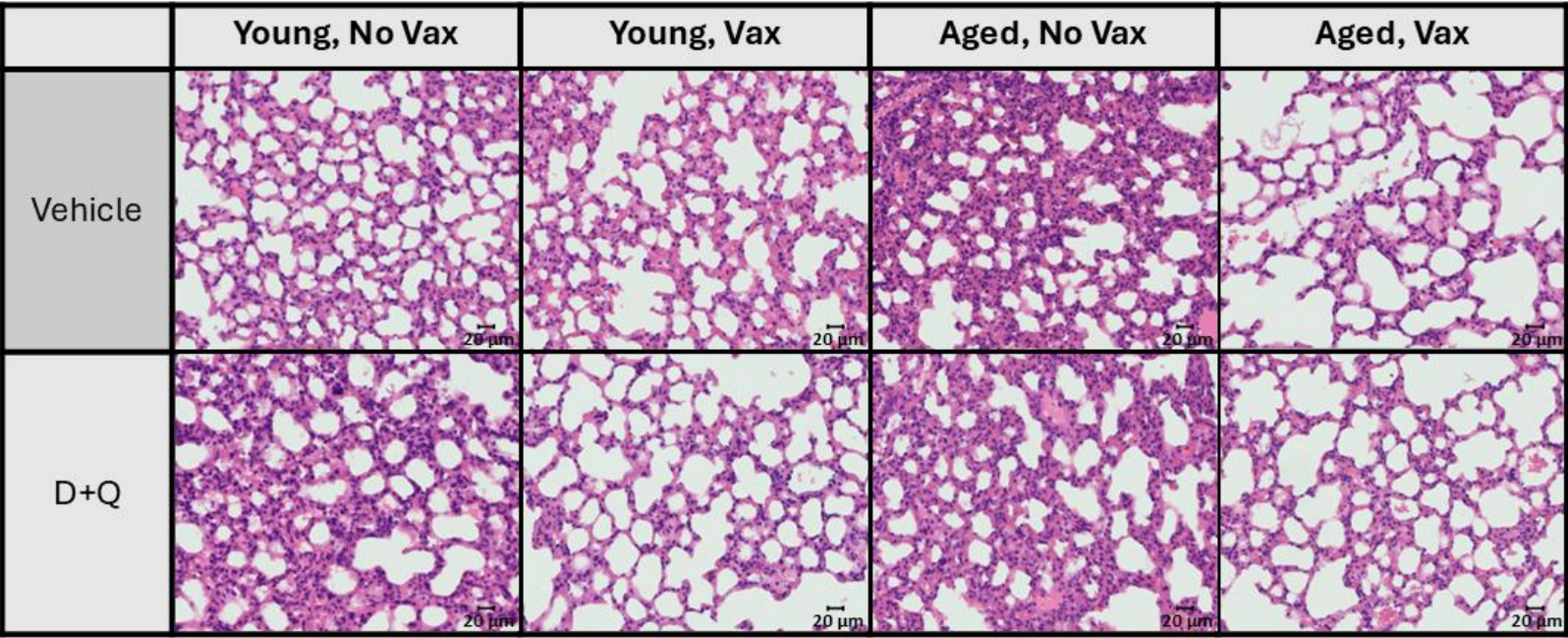

Supplement: Supplementary file 6 — Figure S6: Representative lung histology at 20 days post infection (DPI). Young (3–5 months old) and aged (18–20 months old) C57BL/6JN (B6) mice were treated as in Figure 1A. (A) Representative images of lungs were generated from 5 μm lung sections and stained by hematoxylin and eosin staining (H&E). Data are presented as mean ± standard error of the mean (SEM). Two‐way ANOVA was performed, followed by Šidák's test for multiple comparisons. Results were considered significant at p < 0.05. [file ACEL-25-e70345-s007.pdf]

Supplemental Figure 7

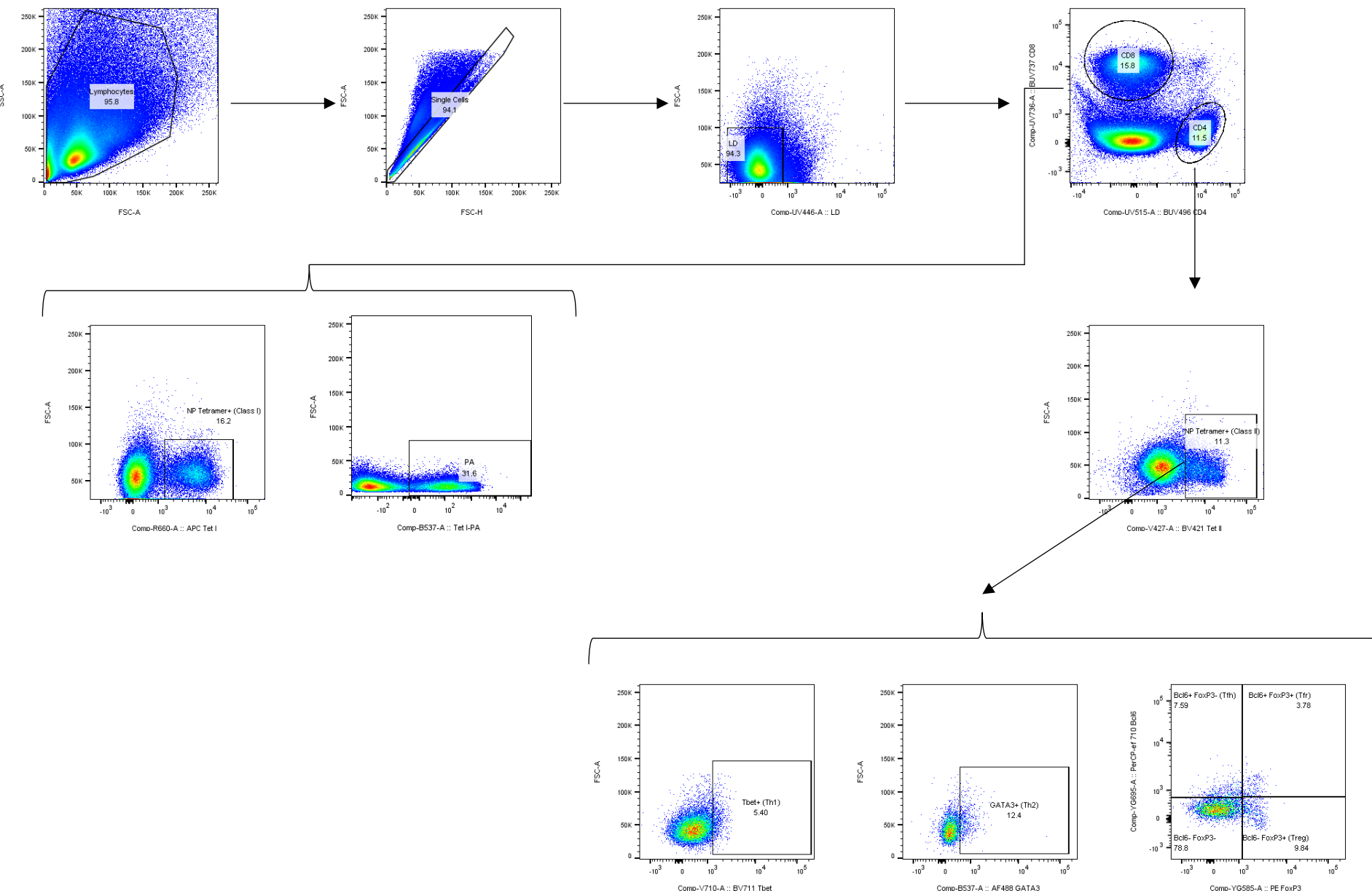

Supplement: Supplementary file 7 — Figure S7: Flow cytometric analysis of T cell phenotypes following flu infection. All T cell phenotyping utilized the gating strategy shown. All cells were gated on Lymphocytes (FSC‐A X SCA‐A), Single Cells (FCS‐H X FCS‐A), Live (Succinimidyl Ester‐A X FCS‐A) and CD4 (CD4+ X CD8‐) and CD8 (CD4‐ X CD8+) T cells. After, within the CD8 T cell population, NP‐specific CD8 T cells were gated using NP366‐374 H‐2Db MHC Class I tetramer (CD8+, NP MHC Class I tetramer+) and PA‐specific CD8 T cells were gated using PA 224–233 H2‐Db MHC Class I tetramer (CD8+, PA MHC Class I tetramer+). NP‐specific CD4 T cells were gated within the CD4 T cell population using NP311‐325 IAb MHC Class II tetramer (CD4+, NP MHC Class II tetramer+). NP‐Specific CD4 T cell subsets were gated within the NP‐Specific CD4 T cell population as follows: NP‐Specific T helper 1 cells (Th1: NP MHC Class II tetramer+, Tbet+), NP‐Specific T helper 2 cells (Th2: NP MHC Class II tetramer+, GATA3+), NP‐Specific T follicular helper cells (Tfh: NP MHC Class II tetramer+, Bcl6+/FoxP3‐), NP‐specific Regulatory T cells (Treg: NP MHC Class II tetramer+, Bcl6‐/FoxP3+), NP‐specific T follicular regulatory (Tfr: NP MHC Class II tetramer+, Bcl6+/FoxP3+). [file ACEL-25-e70345-s008.pdf]
