# Supplementary material for: Senolytic Treatment With Dasatinib and Quercetin Reshapes Influenza‐Specific CD8 T Cell Responses During Infection in Aged, Vaccinated Mice
Source: Aging Cell. 2025 Dec 29;25(1):e70345. doi: 10.1111/acel.70345 (PMC12748518; doi:10.1111/acel.70345)
Supplement: Supplementary file 8 — Table S1: Extended antibody information for flow cytometry experiments. [file ACEL-25-e70345-s001.pdf]

Supplemental Table 1

Specificity (Antigen)	Fluorochrome	Clone	Dilution	Vendor/Supplier
NP311-325 IAb MHC Class II tetramer	BV421	N/A	1:50	NIH Tetramer Core
NP366-374 H-2Db MHC Class I tetramer	APC	N/A	1:50	NIH Tetramer Core
PA 224-233 H2-Db MHC Class I tetramer	BB515	N/A	1:100	NIH Tetramer Core
CD4	BUV496	GK1.5	1:200	BD Biosciences
CD8	BUV737	53-6.7	1:200	BD Biosciences
Tbet (Intracellular)	BV711	4B10	1:100	Biolegend
FoxP3 (Intracellular)	PE	MF-14	1:100	Biolegend
GATA3 (Intracellular)	AF488	16E10A23	1:100	Biolegend
Bcl6 (Intracellular)	PerCP-eF710	BCL-DWN	1:1000	Thermo Fisher
NHS Ester (Succinimidyl Ester)	Alexa Flour 350	-----	1:200	Life Technologies
